# Human immunodeficiency virus, diabetes mellitus and thyroid abnormalities: Should we be screening?

**DOI:** 10.4102/sajhivmed.v21i1.1116

**Published:** 2020-11-09

**Authors:** Somasundram Pillay, Davashni Pillay, Deepak Singh, Romashan Pillay

**Affiliations:** 1 Department of Internal Medicine, King Edward VIII Hospital, Durban, South Africa; 2 Department of Internal Medicine, University of KwaZulu-Natal, Durban, South Africa; 3 Star College Girls Durban, Durban, South Africa; 4 Department of Physics, Durban University of Technology, Durban, South Africa; 5 Clifton College Durban, Durban, South Africa

**Keywords:** patients with diabetes mellitus, HIV infection, thyroid disorders, antiretroviral therapy, patient screening options

## Abstract

**Background:**

Diabetes mellitus (DM) and human immunodeficiency virus (HIV) are associated with thyroid abnormalities. Scarce literature exists on the prevalence of thyroid abnormalities in people living with HIV (PLWH) and DM (PLWHD). Guidelines vary regarding thyroid-stimulating hormone (TSH) screening in PLWH and/or DM.

**Objectives:**

This study describes thyroid abnormalities in PLWHD and HIV-uninfected people living with DM (PLWD).

**Method:**

This was a cross-sectional analysis of demographic, clinical and biochemical data including TSH results of first-visit patients to the Edendale Hospital diabetes clinic between January 2016 and December 2017.

**Results:**

A total of 915 patients were enrolled: 165 PLWHD and 750 PLWD. Overall prevalence of thyroid disorders in PLWD was 8.53% (64/750). The occurrence of ‘total’ thyroid disorders and of ‘subclinical-hypothyroidism’ (SCH) was higher in PLWHD than PLWD (23.03% vs. 8.53% and 20.61% vs. 4%, *p* < 0.001; respectively). People living with HIV and diabetes with thyroid disorders had lower CD4 counts than PLWHD without thyroid disorders (376.08 ± 333.30 vs. 509 ± 341.7 cells/mm^3^; *p* = 0.004). Subclinical-hypothyroidism was more common in patients on antiretroviral therapy [ART] (27/136 [19.85%] vs. 4/27 [14.81%], *p* < 0.001). A significant number of PLWHD acquired HIV before the onset of DM (107/165 [64.85%] vs. 58/165 [35.15%], *p* < 0.001). Patients on ART were more likely to develop DM, OR 2.66 (95% CI 1.11–6.38).

**Conclusion:**

Our study showed an increased prevalence of thyroid disorders (especially SCH) in PLWD and a higher prevalence in PLWHD. Young, overweight, female PLWHD were at risk of SCH. People living with HIV and DM on ART demonstrated an increased prevalence of thyroid dysfunction and poor lipaemic control. The introduction of combined communicable–non-communicable disease clinics might provide an integrated patient screening option.

## Introduction

The emerging burden of diabetes mellitus (DM) and chronic human immunodeficiency virus (HIV) infection will inflict strain on the limited resources of the low- and middle-income countries of Africa. Both conditions have been independently associated with thyroid dysfunction.^1,2,3,4^ Furthermore, thyroid disorders may destabilise the control of both HIV and DM.^1,5^ Thyroid hormones participate in carbohydrate metabolism, insulin secretion and pancreatic function.^6^ Similarly, hypothyroidism is associated with the decreased secretion of insulin from the pancreas.^7^

Shujing et al. showed that thyroid dysfunction, particularly hypothyroidism, is common (33.1%) in people living with HIV (PLWH). This study found that CD4 levels were lower in those with hypothyroidism and that overall biochemical thyroid dysfunction was more frequent in those on antiretroviral therapy (ART) versus those patients who were ART-naïve (39.4% vs. 24.3%, *p* < 0.05, respectively).^2^ Other researchers have also noted an increase of thyroid dysfunction in PLWH. In these studies, few, namely 1% – 2% of PLWH, exhibit overt thyroid disease. However, up to 35% have thyroid-related biochemical abnormalities.^3,4,8,9^ The data have not always been consistent. Jain et al. observed an inverse correlation between CD4 and thyroid-stimulating hormone (TSH) levels and found that progression of HIV was accompanied by a primary hypothyroid state.^5^ In contrast, Lambert noted that both overt and biochemical thyroid dysfunction in PLWH was rare.^10^ Harslof et al. found no difference between treated PLWH and HIV-uninfected persons with regard to the prevalence of hypo- or hyperthyroidism.^11^ Madge et al. has stated that neither HIV nor ART increases the risk of overt or subclinical hypothyroidism (SCH) in PLWH.^12^

People living with HIV who are on ART are now living longer and have become susceptible to chronic conditions such as DM. In some instances, HIV-infected patients have developed insulin resistance resulting from the inflammatory changes that accompany long-term HIV infection and the metabolic toxicities of the early antiretrovirals used at the time.^13,14^

The prevalence of thyroid abnormalities in a United Kingdom population (the Whickham Survey)^15^ was reported to be approximately 6.6%. These data are not specific to PLWD and precede the HIV pandemic by several years. The Colorado (USA) thyroid disease prevalence study of 1995 (*n* = 25 862) found that 9.5% of attendees at a state fair had an elevated TSH test result whilst 2.2% had decreased levels.^16^ The overall prevalence of thyroid abnormalities in PLWD is between 12% and 16%.^1,17,18,19^ Although a Nigerian and an Indian study found higher prevalence rates, namely 46.5% and 30%, respectively, these PLWD subjects may have been non-randomly recruited. Subclinical hypothyroidism was the most frequent diagnosis.^20,21^

Screening guidelines for thyroid disorders in PLWD remain varied globally.^1,21,22,23,24^ Thyroid-stimulating hormone is the most sensitive screening test and establishes the diagnosis of hypo- and hyperthyroidism.^19,25,26^ In the context of HIV, Parsa et al. recommend that TSH be the initial screening tool for the diagnosis of thyroid disorders in PLWH.^27^ Global and South African data on the prevalence of thyroid abnormalities in PLWHD are scarce. International guidelines vary with regard to TSH screening in PLWH and/or PLWHD.

The primary aim of this study was to provide an initial description of some of the thyroid/TSH abnormalities found in South African PLWHD and in those South African PLWD who are HIV uninfected. A secondary aim was to identify the extent, if any, of differences in thyroid/TSH abnormalities between PLWHD and HIV-uninfected PLWD.

## Methods

The visits of all patients attending the Edendale Hospital diabetic clinic are recorded on specially designed data sheets. These were introduced in September 2012 and are completed in triplicate and have been approved by the University of KwaZulu-Natal Biomedical Research and Ethics Committee (BCA 194/95). This ensures that all patients are consulted in a standardised and comprehensive manner. Demographic, clinical and biochemical variables for the patients are captured on these data sheets.

In order to identify concomitant thyroid disorders, TSH tests are performed routinely at all initial clinic visits and the result transferred onto the data sheets. Screening thyroxine (T4) levels were not performed at this clinic because of cost implications. The TSH test, measured in mIU/L, was performed by the National Health Laboratory Services (NHLS) using the Siemens® Avdia Centaur XP analyser. The NHLS’s normal TSH range was 0.35 mIU/L to 5.5 mIU/L. A history of pre-existing thyroid disorders was noted in the medical history and documented as either pre-existing hyper- or hypothyroidism. For this study, patients without a history of a thyroid disorder but with a TSH level < 0.35 mIU/L were classified as having subclinical hyperthyroidism, whilst those patients with a TSH > 5.5 mIU/L and no history of a thyroid disorder were captured as having SCH. Patients with elevated TSH were further subdivided into two groups (5.5–10 mIU/L vs. > 10 mIU/L). This was done as both categories have specific therapeutic implications. Total thyroid disorders were categorised as all patients with either a history of hyper- or hypothyroidism plus all those who were found to have subclinical hypo- or hyperthyroidism. The Bio-Rad D-10 machine (Bio-Rad, USA) was used for the HbA1c analysis. The device and the operator (the NHLS) are National Glycohaemoglobin Standardisation Program (NGSP) accredited. Estimated glomerular filtration rates (eGFR) were calculated by the NHLS using the Modified Diet in Renal Disease (MDRD) formula. The CD4 results are reported in cells/µL or cells/mm^3^.

This is a retrospective cross-sectional study of demographic, clinical and biochemical data, including TSH results, extracted and analysed from the datasheets of all first-visit patients attending the diabetic clinic from 1 January 2016 till 31 December 2017.

Categorical and continuous variables were noted as median and interquartile ranges (25% – 75% IQR). Numbers (*n*) and percentages (%) are provided for categorical variables. Since data was non-parametric, all data were log-transformed. The results shown are back-transformed values. A *p*-value < 0.05 was used as an indicator of significance. Data were analysed by Statistical Package for Social Science (SPSS) version 25 for windows (SPSS Inc., Chicago, IL, USA) and Medcalc (version 19.3.1, Ostend, Belgium).

### Patient and public involvement

Informed consent was not sought as the study is retrospective and all patient-identifying information was anonymised in the study database. Neither patients nor the general public were involved in the design, the operation, the reporting or the dissemination of this research.

### Ethical consideration

Ethics approval for this study was received from the University of KwaZulu-Natal Biomedical Research and Ethics Committee (BE 137/19).

## Results

A total of nine hundred and fifteen (*n* = 915) PLWD were enrolled in the study; *n* = 165 (18.0%) were PLWHD and *n* = 750 (81.9%) were HIV-uninfected PLWD (Table 1). The study revealed that a significant number of PLWHD had acquired HIV *before* the onset of DM (*n* = 107/165, 64.85% [before] vs. *n* = 58/165, 35.15% [after]; *p* < 0.001). After adjusting for age, patients on ART were more likely to develop DM (OR = 2.66 [95% CI 1.11–6.38], *p* = 0.028). The prevalence of ART usage in patients who acquired HIV infection before and after onset of DM was 67.65% (92/136) vs. 32.35% (44/136), respectively. Elevated body mass index (BMI) was associated with increased likelihood of developing DM (OR = 1.136 [95% CI 1.098–1.175], *p* < 0.001). The glycaemic control of both the PLWHD and the HIV-uninfected PLWD was generally suboptimal – median ± IQR HbA1c 9.4% (7–11.2) vs. 9.7% (7.9–11.4), respectively. Nonetheless, a higher percentage of PLWHD achieved optimal glycaemic control, namely HbA1c ≤ 7%, compared to the PLWD (26.43% vs. 15.38%, respectively; *p* < 0.001).

**TABLE 1:** Demographic, laboratory and clinical data of persons living with human immunodeficiency virus and diabetes and human immunodeficiency virus–uninfected people living with diabetes.

Clinical and laboratory characteristics	HIV-infected patients with DM *N* = 165				HIV-uninfected patients with DM *N* = 750				*p*-value Fisher Exact Test, Mann Whitney Test
	*n*	%	Median	IQR	*n*	%	Median	IQR	
**Gender**									
Male	53	32.1	-	-	209	27.87	-	-	0.296
Female	112	67.87	-	-	541	72.13	-	-	0.296
**Type of DM**									
Type 1	22	13.3	-	-	101	13.5	-	-	1.000
Type 2	143	86.6	-	-	649	86.5	-	-	1.000
**Age of patients (years)**	-	-	45	38–54	-	-	58	47–66	< 0.001
**Duration of DM (years)**	-	-	4	1–8	-	-	7	2–15	< 0.001
**BMI (kg/m^2^)**	-	-	30	25–34.25	-	-	31	26–36.48	0.058
HbA1c (%)	-	9.4	7–11.2	-	-	9.7	7.9–11.4	0.095
Total cholesterol (mmol/L)	-	-	4.8	4–5.53	-	-	4.2	3.6–5.1	< 0.001
Triglyceride (mmol/L)	-	-	1.53	0.98–2.63	-	-	1.40	0.97–2.15	0.089
LDL-cholesterol (mmol/L)	-	-	2.62	1.93–3.3	-	-	2.28	1.66–2.97	0.010
HDL-cholesterol (mmol/L)	-	-	1.22	1.01–1.56	-	-	1.18	0.99–1.42	0.049
TSH (mIU/L)	-	-	2.09	1.31–4.45	-	-	1.72	1.15–2.70	< 0.001
**Number of patients with GFR**									
< 60 mL/min	26	18.3	-	-	233	36.46	-	-	< 0.001
> 60 mL/min	116	81.7	-	-	406	63.53	-	-	< 0.001
**Number and percentage of patients with**									
Euthyroid status	127	76.97	-	-	560	74.67	-	-	0.619
Known hypothyroid	1	0.61	-	-	15	2	-	-	0.330
Subclinical hyperthyroidism	3	1.82	-	-	19	2.53	-	-	0.782
Subclinical hypothyroidism	34	20.61	-	-	30	4.0	-	-	< 0.001
Overall thyroid disorders	38	23.03	-	-	64	8.53	-	-	< 0.001
***n* ( % ) of patients on**									
Metformin monotherapy	35	21.21	-	-	129	17.20	-	-	0.220
Insulin monotherapy	39	23.64	-	-	133	17.73	-	-	0.098
Metformin plus other oral antidiabetics	44	26.67	-	-	182	24.27	-	-	0.550
Metformin plus insulin therapy	56	33.94	-	-	333	44.4	-	-	0.015

BMI, body mass index; DM, diabetes mellitus; GFR, glomerular filtration rate; HDL, high-density lipoprotein; HIV, human immunodeficiency virus; IQR interquartile range; LDL, low-density lipoprotein; TSH, thyroid-stimulating hormone.

The prevalence of (total) thyroid disorders was significantly higher in the PLWHD than the HIV-uninfected PLWD, *n* = 38/165 (23.03%) vs. *n* = 64/750 (8.53%), respectively, *p* < 0.001. The principal thyroid disorder was SCH. This was disproportionately represented in the PLWHD (Table 2). A significant percentage of the PLWHD with SCH were female, *p* < 0.001, had Type 2 DM and yet were on insulin monotherapy. The median age of the SCH cohort was older than that of the originator group. The median ± IQR of HIV infection and ART usage in the PLWHD with SCH was 5 (2–9) and 5 (1–9) years, respectively. This group had a median CD4 count of 423 (92.76–888.41) cells/mm^3^. The median CD4 count and inter-quartile range of the entire cohort of PLWHD (*n* = 165) was 446 (155–703) cells/mm^3^. The median CD4 level of PLWHD *with* thyroid disorders was, however, significantly lower than those PLWHD *without* thyroid disorders: CD4 = 376.08 ± 333.30 vs. 509 ± 341.75, respectively; *p* = 0.004.

**TABLE 2:** A subanalysis of subclinical hypothyroidism in people living with human immunodeficiency virus and diabetes versus human immunodeficiency virus–uninfected people living with diabetes.

Variables	PLWHD + Subclinical Hypothyroid group *n* = 34				HIV uninfected PLWD + Subclinical Hypothyroid group *n* = 30				*p*-value (Fisher exact test Mann Whitney Test)
	*n*	%	Median	IQR	*n*	%	Median	IQR	
**Gender**									
Male	10	29.41	-	-	9	30.0	-	-	0.004
Female	24	70.59	-	-	21	70.0	-	-	< 0.001
**Type of DM**									
Type 1 DM	5	14.71	-	-	1	3.33	-	-	0.002
Type 2 DM	29	85.29	-	-	29	96.67	-	-	< 0.001
**Age (years)**	-	-	50.5	42–58	-	-	60	57–68	< 0.001
**Duration of DM**	-	-	4.48	2–8	-	-	9.5	1–17	0.318
**BMI (kg/m^2^)**	-	-	28.9	25–32.75	-	-	36	26–41	0.024
HbA1c (%)	-	-	8	6.2–12.9	-	-	9	7.35–10.85	0.628
Total cholesterol (mmol/L)	-	-	4.8	4.05–5.78	-	-	4.2	4–5.4	0.222
Triglycerides (mmol/L)	-	-	1.69	1.12–2.53	-	-	1.53	1.18–2.42	0.667
LDL-cholesterol (mmol/L)	-	-	2.9	1.7–3.42	-	-	2.0	1.58–2.91	0.239
HDL-cholesterol (mmol/L)	-	-	1.15	0.98–1.52	-	-	1.11	0.91–1.31	0.451
TSH (mIU/L)	-	-	8.85	6.46–11.68	-	-	7.39	6.06–8.82	0.270
**Number and percentage of patients with GFR**									
< 60 mL/min	9	26.47	-	-	19	63.33	-	-	0.142
> 60 mL/min	13	38.24	-	-	10	33.33	-	-	< 0.001
Total Number of Patients with SCH/165 (%)	34	20.61	-	-	30	4.0	-	-	< 0.001
**Number and percentage of patients on**									
Metformin monotherapy	5	3.03	-	-	2	0.27	-	-	0.006
Insulin monotherapy	26	15.76	-	-	28	3.73	-	-	< 0.001
Metformin plus insulin therapy	9	5.45	-	-	11	1.47	-	-	0.022

BMI, body mass index; DM, diabetes mellitus; GFR, glomerular filtration rate; HDL, high-density lipoprotein; HIV, human immunodeficiency virus; IQR interquartile range; LDL, low-density lipoprotein; PLWD, people living with diabetes; PLWHD, people living with human immunodeficiency virus and diabetes; SCH, subclinical-hypothyroidism; TSH, thyroid-stimulating hormone.

People living with HIV and DM versus the HIV-uninfected PLWD were younger, had a shorter duration of DM, a lower but non-significant BMI, *p* = 0.058, and a larger number of individuals with normal renal function namely, GFR > 60 mL/min. The PLWHD group also had significantly higher total LDL and HDL cholesterols (*p* < 0.001, *p* = 0.01, *p* = 0.049, respectively). No such associations characterised patients with subclinical hyperthyroidism (Table 1). The median IQR of the duration of HIV infection and of ART usage by PLWHD was 6 (2–9.25) and 5 (2–9) years, respectively.

Despite the concomitant thyroid disorder, a group of 68 (7.43%) patients continued to receive metformin monotherapy. Less than a percent (7/915, 0.77%) of patients with SCH were on metformin.

Table 3 demonstrates that patients on ART were significantly younger, female and had type 2 DM, and had a higher total, HDL and LDL cholesterol and triglyceride levels; SCH was substantially more common in patients on ART. Patients who were on ART had HIV infection for a longer duration than those who were not. The majority of patients on ART were on a first-line NNRTI-based regimen compared to second-line boosted protease inhibitor-based regimen (11/136, 8.90% vs. 125/136, 91.91%, respectively).

**TABLE 3:** A subanalysis of people living with human immunodeficiency virus on antiretroviral therapy and those not yet started on antiretroviral therapy (antiretroviral therapy-naïve).

Variables	PLWHD on ART (*N* = 136)				PLWHD ART-Naïve (*N* = 27)				*p*-value Mann Whitney Test Fisher Exact Test
	*n*	%	Median	IQR	*n*	%	Median	IQR	
**Gender**									
Male	42	30.88	-	-	10	37.04	-	-	< 0.001
Female	94	69.12	-	-	17	62.96	-	-	< 0.001
**Type of DM**									
Type 1	14	10.29	-	-	8	29.63	-	-	< 0.001
Type 2	122	89.71	-	-	19	70.37	-	-	< 0.001
**Age (years)**	-	-	47	40–55	-	-	42	33–49.25	0.025
**Duration of DM**	-	-	5	2–8	-	-	3	1–9	0.710
**Duration of HIV**	-	-	6.5	3–10	-	-	2	1–3.75	< 0.001
**BMI**	-	-	30	25–34	-	-	32	25.5–39.5	0.215
HbA1c (%)	-	-	9.3	6.95–11.2	-	-	10.2	9.3–11	0.294
Total cholesterol (mmol/L)	-	-	5	4.2–5.6	-	-	4	3.33–4.45	< 0.001
Triglyceride (mmol/L)	-	-	1.7	1.1–2.89	-	-	1.03	0.79–1.2	< 0.001
LDL- cholesterol (mmol/L)	-	-	2.68	2.05–3.36	-	2.09	1.29–2.61	0.014
HDL-cholesterol (mmol/L)	-	-	1.26	1.07–1.65	-	-	1.05	0.92–1.33	0.007
TSH (mIU/L)	-	-	2.08	1.28–4.32	-	-	2.52	1.42–5.45	0.424
CD4 count (cells/mm^3^)	-	-	446	178.5–701.5	-	-	400	99–800	0.945
Number (%) of patients with SCH	-	-	27	19.85	-	-	4	14.81	< 0.001
Subclinical Hyperthyroidism *n* (%)	-	-	3	2.21	-	-	-	-	< 0.001

ART, antiretroviral therapy; BMI, body mass index; DM, diabetes mellitus; HDL, high-density lipoprotein; HIV, human immunodeficiency virus; IQR interquartile range; LDL, low-density lipoprotein; PLWHD, people living with human immunodeficiency virus; SCH, subclinical hyperthyroidism; TSH, thyroid-stimulating hormone.

Whilst we found an inverse correlation between CD4 counts and TSH levels within the entire PLWHD cohort, this was not significant (*r* = ‒0.084. Pearson’s correlation). This inverse correlation was present in patients on ART and those that were ART-naïve (*r* = 0.083 vs. 0.091, respectively).

Subdivision of the cohorts into those with a moderately high TSH (5.5–10 mIU/L) and those with an extremely high TSH (> 10 mIU/L) revealed that the two groups were evenly matched for most demographic, clinical and biochemical variables. A significantly greater number of patients in the TSH (5.5–10) cohort had suboptimal glycaemic control (*p* = 0.009). A substantial number of patients with SCH had TSH values in the range of 5.5–10 mIU/L (*p* = 0.006). The shorter duration of both HIV and DM in those with very high TSH levels suggests a group who are at risk of a more precipitous course of SCH. Women outnumber men in this ‘more severe’ group, and this particularly so in obese women (Table 4).

**TABLE 4:** Stratification according to thyroid-stimulating hormone value.

Variables	TSH 5.5–10 mIU/L (*n* = 46)				TSH > 10 mIU/L (*n* = 25)				*p*-value Mann Whitney Test Fisher Exact Test
	*n*	%	Median	IQR	*n*	%	Median	IQR	
**Gender**									
Male	15	32.6	-	-	5	20	-	-	0.287
Female	31	67.4	-	-	20	80	-	-	0.287
**Type of DM**									
Type 1	4	8.7	-	-	2	8	-	-	> 0.05
Type 2	42	91.3	-	-	23	92	-	-	> 0.05
**Age (years)**	-	-	59.5	44–64	-	-	58	51–64.25	0.947
**Duration of DM (years)**	-	-	8	2–15	-	-	6	1.68–10.24	0.406
**Duration of HIV (years)**	-	-	5.5	2.5–9	-	-	2.45	1–9	0.236
**Duration of ART (years)**	-	-	5.5	2–9	-	-	2	1–9	0.264
**BMI (kg/m^2^)**	-	-	29.5	25–38	-	-	33	29–37	0.155
HbA1c (%)	-	-	9.3	7.53–12.8	-	-	7.15	6.16–10.5	0.074
Total Cholesterol (mmol/L)	-	-	4.6	4–6	-	-	4.6	3.85–5.38	0.332
Triglyceride (mmol/L)	-	-	1.58	1.14–2.52	-	-	1.69	1.27–2.15	0.852
LDL- cholesterol (mmol/L)	-	-	2.57	1.72–3.4	-	-	2.22	1.67–3.02	0.580
HDL-cholesterol (mmol/L)	-	-	1.21	1–1.39	-	-	1.08	0.91–1.38	0.339
TSH (mIU/L)	-	-	6.77	5.99–7.6	-	-	14	11.56–20.36	< 0.0001
CD4 count (cells/mm^3^)	-	-	453	121.75–999.75	-	-	214.77	78.04–646.37	0.209
**Number (%) of patients with**									
SCH	43	93.48	-	-	21	84	-	-	0.006
**Number (%) of patients with HbA1c**									
≤ 7%	8	22.9	-	-	9	45	-	-	0.808
> 7%	27	77.1	-	-	11	55	-	-	0.009

ART, antiretroviral therapy; DM, diabetes mellitus; HDL, high-density lipoprotein; HIV, human immunodeficiency virus; IQR interquartile range; LDL, low-density lipoprotein; SCH, subclinical hyperthyroidism; TSH, thyroid-stimulating hormone.

## Discussion

Diabetes mellitus and HIV infection pose a long-term global healthcare and fiscal problem for Africa. Both are independently associated with thyroid abnormalities. Data on the global prevalence of thyroid disorders in PLWHD are limited, with African data even more scarce.

Our study showed that within the cohort of HIV-uninfected PLWD, thyroid disorders were more common in female type 2 PLWD. These results correlated with those of Udiong et al. in Nigeria,^20^ but not so with Perros et al., whose data indicated that thyroid abnormalities were more common in type 1 DM.^1^ The subject base of our diabetic clinic reflects a preponderance of adult type 2 PLWD. Most young type 1 PLWD are cared for at the local tertiary hospital. The PLWHD had better glycaemic control, were younger, had DM for a shorter duration, had lower BMI and preserved renal function but had higher lipaemic levels than their HIV-uninfected PLWD counterparts, whilst the group of patients who were ART-naïve had a shorter duration of HIV infection and a decreased prevalence of SCH. The PLWHD may offer an opportunity to get ahead on DM and possibly thyroid management if integrated into ART control.

Twenty-three per cent (23.03%) of the PLWHD in this study had thyroid disorders, most notably, SCH in 20.61%. These results were similar to what Shujing et al.,^2^ Madeddu et al.,^4^ Brockmeyer et al.^28^ and Silva et al.^9^ showed in their studies. People living with human immunodeficiency virus and diabetes were mostly female, older, had type 2 DM, had increased BMI and were mainly on insulin monotherapy when compared to PLWHD. Weight gain in PLWHD may be a marker of thyroid dysfunction and needs to emphasised in patient evaluation.

Discussion on the implications of SCH and its management is ongoing. The risk of SCH is increased in PLWD, PLWHD and those on ART.^3,4,8,29,30^ Han et al. concluded in their meta-analysis that PLWD with SCH had increased diabetes-related complications in the form of nephropathy, retinopathy, peripheral arterial disease and peripheral neuropathy.^31^ The most important consequence of SCH remains progression to overt hypothyroidism (ranging from 3% to 18% annually).^32,33,34,35,36^ The Rotterdam study demonstrated an association between patients with SCH, aortic calcification and myocardial infarction and identified SCH as an independent risk factor for myocardial infarction.^37^ Other observational studies have also found associations between SCH and coronary artery disease.^38,39,40^ This association was, however, not found in the Wickham study.^41^ Haentjens et al. in their analysis of seven cohort studies found that there was an increased risk of all-cause mortality in patients with SCH, more so in patients with co-morbid conditions.^42^ We show that SCH is associated with hyperlipidaemia. Undiagnosed SCH may affect metabolic control and worsen cardiovascular risk in PLWD.^43,44^ Baseline TSH screening would help identify those patients who are at higher cardiovascular risk that need closer monitoring.

We demonstrated that over one-third (25/71, 35.21%) of our patients with SCH had TSH levels of > 10 mIU/L, and this was predominantly in obese female patients with a short duration of DM and HIV infection. Most authors agree that patients with a TSH > 10 mIU/L should be initiated onto levothyroxine therapy (LT).^45,46,47^ However, 64.78% (46/71) of our study SCH patients had TSH levels of between 5.5 and 10 mIU/L, which poses a therapeutic conundrum to the attending physician. Levothyroxine therapy in this group of patients is still very much debated in literature. Both Surks et al. and Kong et al. found no benefit in initiation of LT in this TSH range,^48,49^ whilst McDermott et al., Kadiyala et al. and Fatourechi advocated that patients with SCH with co-morbid cardiovascular conditions be treated with LT.^45,46,50^ It must be borne in mind that these studies were conducted in HIV-uninfected patients.

Thyroid dysfunction in the form of SCH was found to be significantly more common in patients on ART therapy in our study (22.06%). Although less common, it must be noted that thyroid disorders were also prevalent in patients who were ART-naïve (14.81%). This finding has also been demonstrated by Jain et al. in their study conducted in India.^5^ Our study also found that the CD4 counts were significantly lower in patients with combined HIV infection and thyroid disorders. The link between being on ART, having lower CD4 counts and thyroid dysfunction was also found in the study conducted by Shujing et al.^2^ and serves to highlight the importance of screening for thyroid disorders in both HIV clinics and in combined communicable–non-communicable disease (HIV-DM) clinics. In our study, patients on ART had higher levels of total cholesterol, triglycerides and LDL cholesterol and had HIV for a longer duration than their ART-naïve counterparts, illustrating the complex interactions between ART and increased cardiovascular risk.

Interestingly, we found that a significant number of HIV-infected patients developed DM after the onset of HIV infection. The aetiology of DM in the setting of HIV can be attributed to either the inflammatory milieu associated with the HIV infection itself, the ART or increased patient longevity.^14^ This finding underscores the importance of screening for DM in HIV clinics.

Studies have found that the occurrence of DM in the presence of HIV infection is usually due to insulin resistance rather than insulin deficiency and therefore is more likely to be type 2 in nature.^13^ Metformin use for the treatment of DM in the presence of HIV has been associated with increased risk of developing diarrhoea and may cause lactic acidosis in patients with renal failure. The advantages of metformin use in PLWHD remains that it is an insulin sensitiser, is cost-effective and can assist with weight loss.^51^ Insulin remains the treatment of choice in PLWHD and is endorsed by the American Association of Clinical Endocrinologists. The benefits of using insulin is that it does not cause gastrointestinal side effects and has no interactions with ART; however, insulin usage does pose increased risks of hypoglycaemia and requires safe disposal of needles.^52^ Metformin use in patients is discouraged in PLWD and thyroid dysfunction as in-vitro studies using metformin have demonstrated that metformin has inhibitory effects on cell proliferation.^53^ The majority (81.82%) of our PLWHD were still on metformin therapy (either monotherapy or in combination with other oral antidiabetic therapies or with insulin). However, within the group of HIV-uninfected PLWD and thyroid dysfunction, this message of the decreased use of metformin seemed to have been heeded, as the majority of these patients were on insulin therapy as compared to metformin (6.93% vs. 4.26%, respectively). This trend of using insulin instead of metformin monotherapy was also observed in the majority of patients with HIV infection and thyroid disorders (17.58% vs. 10.31%, respectively).

The cohort comprising the PLWHD but with no thyroid disorders were generally female, had type 2 diabetes, were younger, had a shorter duration of DM and a lower BMI with a significant percentage having glomerular filtration rates of > 60 mL/min. Pillay et al. have shown previously that PLWHD were at increased risk for nephropathy.^54^ Our findings of higher GFR and a greater percentage of patients achieving target HbA1c can be explained by the fact that this cohort were younger, not obese and only had DM for a short duration. Another important study finding was that this cohort had significantly higher total cholesterol and triglycerides when compared to their HIV-uninfected counterparts. Both DM and HIV are predisposing factors for cardiovascular morbidity and mortality.^43,44,55^ Our findings of elevated total cholesterol and triglyceride in this cohort will serve to further increase their cardiovascular risk.

Although not statistically significant, glycaemic control was poorer in the PLWHD compared to the PLWD with regards to those:

- with overall total thyroid disorders
- with SCH
- on ART

Future prospective studies are needed to determine the extent of this glycaemic difference between these groups of patients which may aid in providing another therapeutic avenue in improving overall diabetes control.

## Strengths and limitations of study

- Results of this study will help strengthen the case for evaluation of thyroid disorders in PLWD but more especially in PLWHD.
- The limitations of this study include that we only used a single TSH level for diagnosis of thyroid dysfunction. Assay-related analytic errors and transient thyroid dysfunction secondary to drugs like lithium and amiodarone were not assessed.
- People living with HIV are at heightened risk of developing the euthyroid sick syndrome. During periods of recovery from acute illness, the TSH might be increased in this condition and may result in an overdiagnosis of SCH.
- Another limitation included that no T4 or T3 testing was performed in patients with low or high TSH. As this was a retrospective first visit study, we were able only to collect TSH data that were gathered during screening on first visit.
- A further limitation remains that no viral load and effectiveness of ART data was available to assess if patients were taking their medication and whether ART influenced the outcome.

## Conclusion

Data on thyroid disorders in PLWHD are scarce. Our study has shown an increased prevalence of thyroid disorders (most notably SCH) in HIV-uninfected PLWD and an even higher prevalence in PLWHD.

Young overweight women who are HIV infected and have DM seem to be particularly at risk of SCH. This is a ‘key’ population group that should be evaluated in all HIV clinics for thyroid disorders.

People living with human immunodeficiency virus and diabetes on ART were shown to have an increased prevalence of thyroid dysfunction with poorer lipaemic control. This particular group of patients on ART need to be regularly screened for the aforementioned cardiovascular complications. The introduction of combined communicable–non-communicable disease clinics might provide an integrated option for these patients.
